# Integrated Biochemical and Ultrastructural Responses of *Tanacetum vulgare* L. to Multi-Metal Stress

**DOI:** 10.3390/plants15071112

**Published:** 2026-04-03

**Authors:** Ilya Alliluev, Natalia Chernikova, Victoria Kazachkova, Irshad Ahmad, Aleksei Fedorenko, Vladislav Popov, Artem Babenko, Victor Chaplygin, Saglara Mandzhieva, Tatiana Minkina

**Affiliations:** Academy of Biology and Medicine, Southern Federal University, Rostov-on-Don 344090, Russia; alliluev@sfedu.ru (I.A.); nat.tchernikova2013@yandex.ru (N.C.); vkazachkova@sfedu.ru (V.K.); afedorenko@mail.ru (A.F.); vpop@sfedu.ru (V.P.); arba@sfedu.ru (A.B.); chaplygin@sfedu.ru (V.C.); msaglara@mail.ru (S.M.); tminkina@mail.ru (T.M.)

**Keywords:** rhizosphere, antioxidant defense system, bioaccumulation, cell ultrastructure, heavy metal, oxidative stress, soil pollution

## Abstract

Coal combustion at power stations is a significant source of heavy metal accumulation in plants and soil, posing risks to ecosystems and human health. The objective of the study was to investigate the adaptive strategies of common tansy (*Tanacetum vulgare* L.) exposed to heavy metal pollution in the impact zone of the Novocherkassk State Power Station (Russia). In the impact zone, soil concentrations of Cd, Pb, Ni, Cr, Cu, Zn, and Mn exceeded background levels by 1.4–8.2 times. An analysis of heavy metal translocation revealed selective accumulation mechanisms. The Cd translocation factor increased by 5.6-fold and Pb by 6-fold, correlating with a 14- and 22-fold enrichment of mobile compounds of Cd and Pb in the rhizosphere. *T. vulgare* demonstrated a coordinated antioxidant response: the activity of superoxide dismutase (+27%), guaiacol peroxidase (+375%), catalase (+348%), as well as the content of glutathione (+11%), increased in shoots. However, the polyphenol content in the shoots decreased by approximately 22%. Despite severe ultrastructural damage, *T. vulgare* maintained high biomass productivity. This selective translocation phenotype, combined with high biomass productivity, makes the species a promising candidate for the phytoremediation of coal-contaminated soils.

## 1. Introduction

Coal is widely used in power stations for electricity generation [1,2]. According to the International Energy Agency, coal accounted for about 35% of the global energy generation in 2024 [3]. Coal combustion negatively affects ecosystems and leads to the release of particulate matter, nitrogen oxides, sulfur dioxide, and heavy metals (HM) into the environment [4,5,6]. The Novocherkassk power station is the main power station in the Rostov region (Russia) and a major source of HM pollution. HM contents exceeded the maximum permissible levels in the impact zone of the Novocherkassk power station [7]. The particulate atmospheric deposition at the site contained 13 times more Zn, 7–8 times more Pb and Cu, 1.5–2 times more Ni, Cr and Cd than the regional background [8]. Plants accumulate HM in large quantities in their tissues, and these metals subsequently enter the food chain [9].

The rhizosphere, the zone of intensive microbial activity directly adjacent to plant roots, represents an additional layer of defense against HM toxicity. This biogeochemical interface regulates the mobility and bioavailability of HM in contaminated soils through several key processes. These include biosorption, bioaccumulation, chelation by organic ligands, and redox transformations. Collectively, these processes reduce the bioavailable metal fraction in the soil [10]. Plants colonizing anthropogenically disturbed soils typically exhibit an enhanced tolerance to complex HM contamination, and this adaptive capacity largely depends on the functional activity of rhizosphere microbial communities, which decrease contaminant bioavailability and modulate the redox status of the rhizosphere [11].

Previous studies have identified the mechanisms through which plants absorb and accumulate HM in their organs [12,13,14]. HM stress leads to an excessive production of reactive oxygen species (ROS) [15,16]. A low concentration of ROS acts as a signal and triggers a stress response in plants, while a high concentration causes damage and programmed cell death [17]. HM such as Cu and Cr catalyze the generation of ROS via Fenton-type reactions, Pb and Cd impair the plant’s capacity to scavenge ROS [18]. The overproduction of ROS damages cell membranes, nucleic acids, and proteins and results in cell death [19]. HM stress results in alterations in gene expression and metabolic pathways [20,21,22]. HM contamination affects the enzyme activity involved in the biosynthesis of bioactive compounds and contributes to alterations in secondary metabolite concentrations [23,24,25]. Excessive concentrations of Mn, Zn and Ni disturb photosynthesis, leading to reduced carbon fixation [26]. The antioxidant defense system is crucial for protecting plants from oxidative stress [27,28]. Enzymatic antioxidants form the first line of defense against ROS. The enzymatic components associated with the defense against ROS are superoxide dismutase (SOD), catalase (CAT), and peroxidase (POD) [29]. Non-enzymatic antioxidants provide complementary protection by scavenging excess ROS and stabilizing cellular structures [30,31,32]. Many of these low-molecular-weight compounds, such as phenolic compounds and flavonoids, are derived from secondary metabolic pathways and operate alongside enzymatic systems to maintain cellular homeostasis under HM stress [33]. HM toxicity also affects plant microstructure, resulting in cell wall thickening and degeneration, changes in the number of tracheid and vessel elements in the vascular cylinder, and the rupture of parenchyma tissue [34].

Common tansy (*Tanacetum vulgare* L., Asteraceae) is a perennial herbaceous medicinal plant, native to Asia and Europe. *T. vulgare* is widely distributed in temperate climates, and is tolerant to a complex of unfavorable conditions, which indicates its high ecological plasticity in different environmental conditions [35]. *T. vulgare* has been shown to be a bioindicator of trace metal contamination in naturally colonized open pit lignite mines [36]. *T. vulgare* spontaneously colonizes anthropogenically disturbed soils enriched in HM. The adaptive potential of this specie is supported by a well-developed root system and the intensive synthesis of secondary metabolites with antioxidant properties [35]. Studying the adaptation of native species to unfavorable edaphic conditions is of practical importance in the development of technologies for the phytoremediation of anthropogenically transformed soils [37]. However, the functional role of the rhizosphere in modulating HM bioavailability and plant tolerance under field conditions in coal power station impact zones has not been systematically characterized. Furthermore, a comprehensive, integrated assessment linking biochemical responses to ultrastructural changes and organismal-level survival remains lacking. In this context, the objective of this study was to characterize the adaptive strategies of *T. vulgare* and the role of the rhizosphere under HM pollution in the impact zone of the Novocherkassk power station.

## 2. Results

### 2.1. Content of HM in Soil

**Figure 1:**
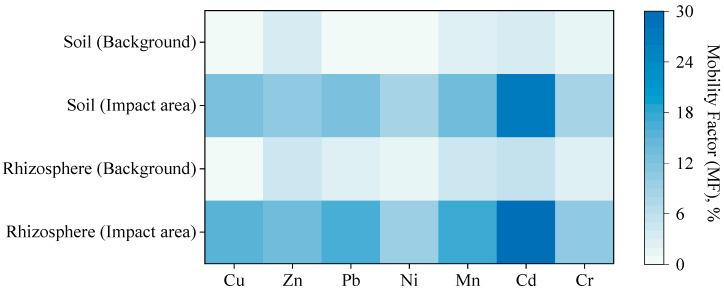
Heatmap showing the mobility factor (MF) of HM in soil and rhizosphere samples. Note: Background—Persianovskaya protected steppe; Impact area—impact zone of the Novocherkassk power station. The MF was calculated as the relative abundance of mobile forms to the total content (C_mobile forms_/C_total_, %).

The total concentration of HM in the soil, as well as the content of mobile forms in the soil and rhizosphere of *T. vulgare,* was markedly higher in the impact zone of the Novocherkassk power station compared with the background site (Table 1). At the Persianovskaya protected steppe, the total HM content decreased in the order Mn > Cr > Zn > Ni > Pb ≥ Cu > Cd. In soils near the Novocherkassk power station, the HM concentrations decreased in the order Mn > Zn > Cr > Cu > Pb > Ni > Cd. The content of mobile forms in the background soil was 0.2–4.9% of the total content and 1.3–29.2% in the contaminated soil. The rhizosphere of *T. vulgare* was characterized by a high bioavailability of metals compared with the soil, especially Cd, Mn and Pb (Figure 1).

### 2.2. HM Content in T. vulgare and Translocation

HM concentrations in *T. vulgare* were significantly higher at the polluted area near the Novocherkassk power station than in the background area (Table 2). Metal accumulation showed clear organ-specific patterns. Cu, Zn and Ni predominantly accumulated in the roots, while Pb, Mn, Cd and Cr were present in the shoots in higher concentrations. In the impact area near the coal power station, the Cu level in the roots and shoots of tansy was increased by 1.7 times, Zn by 1.5 times, Pb by 2.4 times, Ni by 1.9 times, Mn by 1.4 times, Cd by 4.2 times and Cr by 1.8 times, relative to the background soil.

Calculated bioconcentration and translocation factors revealed contrasting metal uptake patterns depending on the pollution level (Table 3). At the background site, *T. vulgare* predominantly retained metals in the roots, displaying TF values below 1.0 for Pb, Cu, Ni, and Zn. The only exceptions were Mn and Cr, which were actively transported to the shoots. In the impact zone, the translocation pattern shifted significantly, particularly for non-essential toxic elements. The most pronounced change was observed for Cd, where the TF increased from 0.8 to 4.5. Similarly, Pb mobility increased strongly, shifting from pronounced root retention to active shoot accumulation. For all metals studied, BCF_root_ and BCF_shoot_ remained below 1 (Table 3).

### 2.3. Biochemical Parameters

#### 2.3.1. Malondialdehyde and Proline Content

**Figure 2:**
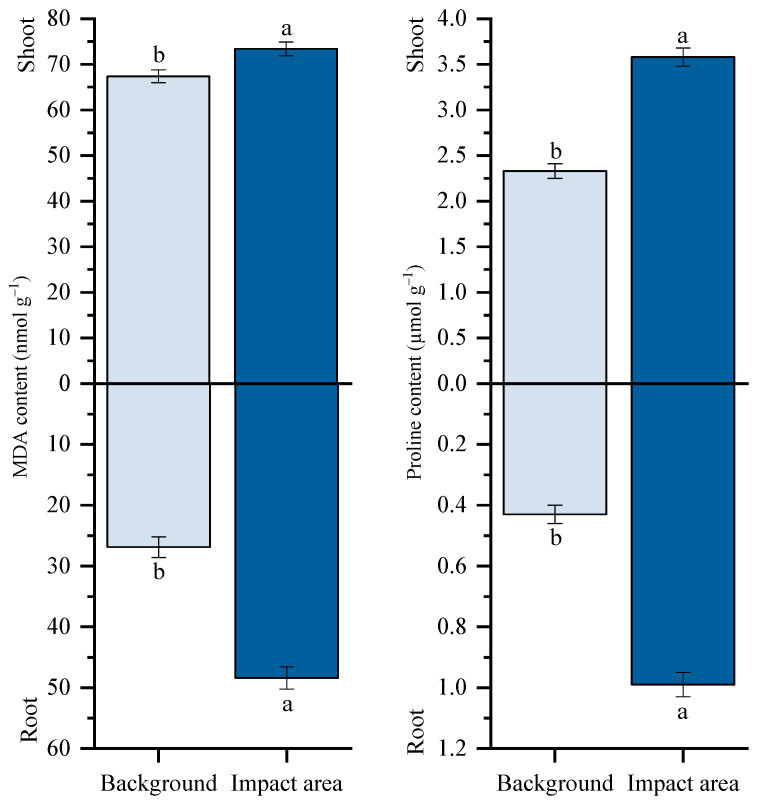
Malondialdehyde (MDA) and proline content in the shoot and in the root of common tansy (*Tanacetum vulgare* L.). Note: Background: Persianovskaya protected steppe; Impact area: impact zone of the Novocherkassk power station. Vertical bars represent the standard error of the mean. Lowercase letters indicate significant differences at *p* ˂ 0.05 (*t*-test).

Multi-metal exposure elicited pronounced oxidative stress in *T. vulgare*, as indicated by elevated MDA levels and concurrent proline accumulation. MDA content was consistently higher in shoots than in roots at both sites. However, plants collected from the impact area exhibited higher MDA levels compared with the background site, with increases of approximately 9% in shoots and 80% in roots (Figure 2). In parallel, proline accumulation was significantly enhanced under multi-metal stress. Proline content increased by 54% in shoots and by 131% in roots, relative to the background site, indicating an active osmoprotective response.

#### 2.3.2. Antioxidant Enzyme Activity

**Figure 3:**
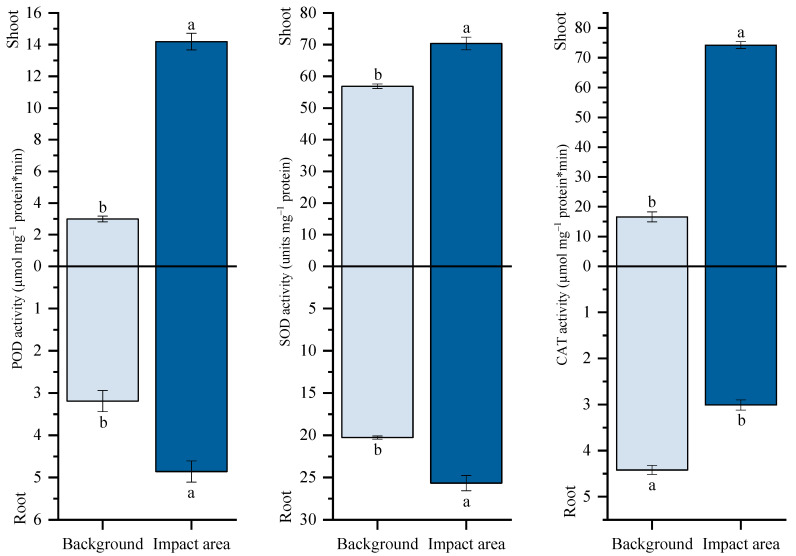
Guaiacol peroxidase (POD), superoxide dismutase (SOD), and catalase (CAT) activity in the shoot and in the root of *T. vulgare*. Note: Background: Persianovskaya protected steppe; Impact area: impact zone of the Novocherkassk power station. Vertical bars represent the standard error of the mean. Lowercase letters indicate significant differences at *p* ˂ 0.05 (*t*-test).

Activities of antioxidant enzymes were markedly altered under multi-metal exposure (Figure 3). SOD activity increased in both shoots and roots by 24% and 27% compared with plants grown in the Persianovskaya protected steppe. POD exhibited the strongest response, with activity increasing by 375% in shoots and by 53% in roots. In contrast, the activity of CAT increased substantially in shoots by 348% but decreased in roots by 32%.

#### 2.3.3. Non-Enzymatic Antioxidants Content

**Figure 4:**
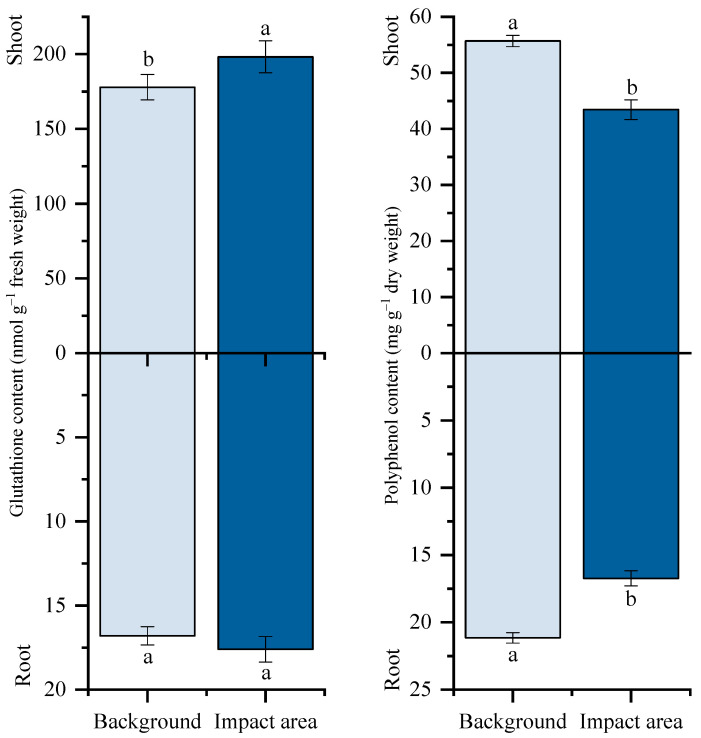
Glutathione and polyphenol content in the shoot and in the root of *T. vulgare*. Note: Background: Persianovskaya protected steppe; Impact area: impact zone of the Novocherkassk power station. Vertical bars represent the standard error of the mean. Lowercase letters indicate significant differences at *p* ˂ 0.05 (*t*-test).

Non-enzymatic antioxidant pools in *T. vulgare* were also modified, with contrasting responses of phenolic compounds and glutathione (Figure 4). At the background site, polyphenol concentrations were higher in shoots than in roots, reaching 55.69 and 21.15 mg g^−1^ in dry weight, respectively. In plants collected from the impact zone, polyphenol content declined significantly in both organs, decreasing by approximately 22% in shoots and 21% in roots compared with the background site. In contrast, glutathione content increased substantially under multi-metal exposure. In shoots, glutathione levels increased by 11%, relative to background conditions, while a moderate increase of 17% was observed in roots.

### 2.4. Morphological and Ultrastructural Findings

#### 2.4.1. Root Morphology and Ultrastructure

**Figure 5:**
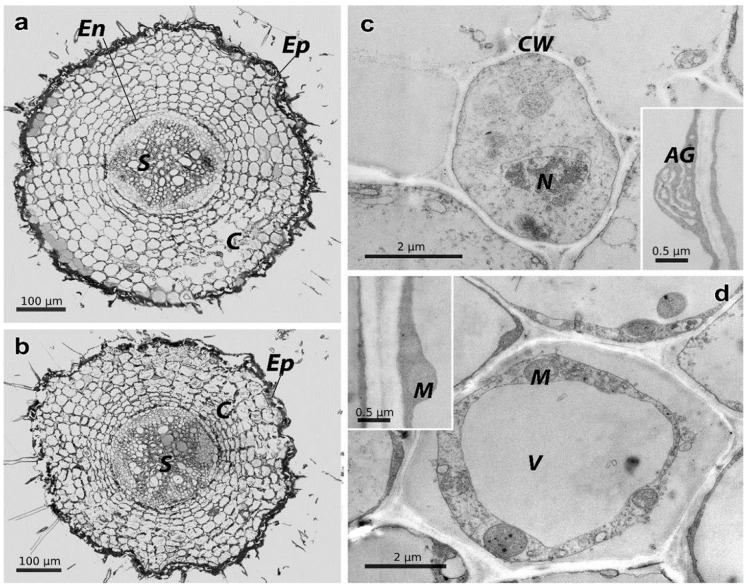
Light-optical and electron microscopy of *T. vulgare* roots. Letters indicate experimental variants: (**a**)—Persianovskaya protected steppe, (**b**)— impact zone of the Novocherkassk power station. Key: **Ep**—epiblema, **En**—endoderm, **C**—root cortex, **S**—stele (central cylinder). TEM image of common tansy root cells. (**c**)—Persianovskaya protected steppe, (**d**)—Novocherkassk power station. Key: **AG**—Golgi apparatus, **M**—mitochondria, **V**—vacuole, **N**—nucleus, **CW**—cell wall.

Control roots had undergone secondary development with normal tissue architecture (Figure 5a). The epiblem retained a distinct structure (23,000 ± 1150 µm^2^ area) with compactly organized cells. The primary cortex (139,307 ± 6965 µm^2^ area) was clearly differentiated and consisted of densely packed (2083 ± 62 cells per 1 mm^2^), evenly shaped and 329 ± 53 µm^2^ sized parenchymatous cells. The central cylinder (40,141 ± 1405 µm^2^ area) was well defined; the vascular-fibrous bundle was already partially replaced by a secondary xylem and phloem. The sclerenchyma of the pericyclic zone consisted of small, densely packed cells with thickened cell walls. Pericyclic cells were larger than the surrounding cells, elongated, and arranged in a single row. Xylem vessels had normal wall thickness and open lumens, while phloem elements were isolated and active. Roots were highly differentiated and functionally active (Figure 5c). In the parenchyma cells of the primary cortex, the cytoplasm was localized along the cell walls as a thin rim, thickening at the locations of organelles. The central vacuole occupied most of the cell volume. Organelles were evenly distributed in areas of cytoplasmic thickening. Mitochondria had a light matrix with clearly defined cristae. The endoplasmic reticulum was well developed.

Roots from the contaminated area retained their secondary structure but displayed substantial structural alterations (Figure 5b). The epiblem with an area of 30,345 ± 1517 µm^2^ occupied the same volume (11–12%) of the total cross-sectional area as in the control. The primary cortex (176,132 ± 8807 µm^2^ area) occupied 69% of the total root section and was composed of loose cells that were 363 ± 98 µm^2^ in size, with irregular shapes in places. The intercellular space was dilated (1941 ± 58 cells per 1 mm^2^). The central cylinder was significantly smaller in size (37,585 ± 1315 µm^2^ area) than in the background conditions. The xylem and phloem had thickened cell walls and were less symmetrically arranged (Figure 5d). The cytoplasm in the parenchyma cells of the primary cortex (as in the control cells) was distributed along the cell walls as a thin rim, with occasional thickening at the sites of organelles. However, organelles in many cells of plants from the contaminated site were impaired: the mitochondria were reduced in size and the cristae were poorly defined. The central vacuoles were enlarged, often occupying almost the entire cell volume. Crystalline deposits were observed within the vacuoles and the endoplasmic reticulum appeared fragmented.

#### 2.4.2. Leaf Morphology and Ultrastructure

**Figure 6:**
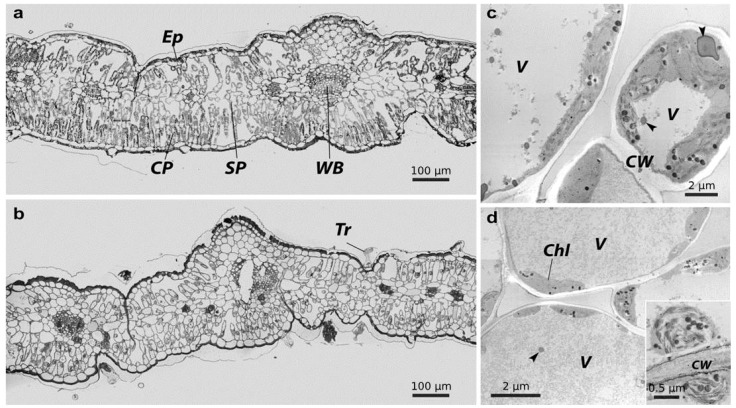
Light-optical and electron microscopy of *T. vulgare* leaf. Letters indicate experimental variants: (**a**)—Persianovskaya protected steppe, (**b**)—impact zone of the Novocherkassk power station. Key: **Ep**—epidermis, **CP**—columnar parenchyma, **SP**—spongy parenchyma, **WB**—vascular bundle, **Tr**—trichome. TEM image of tansy leaf cells. (**c**)—Persianovskaya protected steppe, (**d**)—Novocherkassk power station. Key: **Chl**—chloroplast, **V**—vacuole, **CW**—cell wall, **arrow**—lipid droplet.

Control leaves displayed a typical anatomy, with a single-layered epidermis, well-differentiated mesophyll (palisade and spongy parenchyma) and organized vascular tissue (Figure 6a). The epidermis formed a single-row layer of cells with a cuticle, the surface of which contained structures that synthesized essential oils, including glandular trichomes. The mesophyll consisted of a palisade layer formed by compactly arranged cells and a spongy layer with a looser structure. The leaf vascular system (9623 ± 251 area µm^2^) was fully formed, with vessels and tracheid having dense, smooth walls. Leaf venation was characterized by a clear organization, and cells around the vascular tissues had uniform wall thicknesses. Chlorenchyma cells were developed and characterized by a uniform distribution of plastids (12 ± 4 columnar and 5 ± 2 spongy plastids per cell) in the cytoplasm (Figure 6c). Thylakoid grana in the plastids were arranged in an orderly manner along the long axis. Plastoglobuli were few, and starch grains were small and evenly distributed in the stroma. The stomatal density was 5 ± 2 per mm^2^.

Leaves from the contaminated area showed substantial epidermal damage compared with the control. The cuticle exhibited discontinuities and irregular deposition, and underlying epidermal cells displayed aberrant wall architecture, variable thicknesses and local degradation (Figure 6b). Glandular trichomes showed signs of deformation. Mesophyll densities varied: the palisade layer was poorly developed, and the cells were sparsely arranged. In the spongy layer, cells were enlarged, closely contacting each other, and the intercellular space (14,387 ± 360 µm^2^) was undeveloped. Vascular tissues (9770 ± 310 area µm^2^) in the veins were damaged, vessels showed signs of deformation, and the tracheid structure was disorganized in places. Venation was uneven, and local cell death was sometimes observed around vessels. Signs of moderate stress were observed in chlorenchyma cells (Figure 6d): thylakoids in plastids were disorganized, the number of plastoglobuli and lipid droplets increased, and starch grains were large. Lipid bodies were present in the cytoplasm and central vacuole. Stomatal density was decreased from 5 ± 2 per mm^2^ to 3 ± 3 per mm^2^. The parenchyma area was significantly reduced, with columnar tissue declining from 50,911 ± 677 µm^2^ to 36,928 ± 491 µm^2^, and spongy tissue from 10,073 ± 127 µm^2^ to 8194 ± 105 µm^2^. Concurrently, the average cell size also decreased significantly in both tissues: columnar cells from 486 ± 121 µm^2^ to 311 ± 125 µm^2^ and spongy cells from 496 ± 154 µm^2^ to 333 ± 146 µm^2^.

## 3. Discussion

Emissions from the Novocherkassk power station resulted in marked HM accumulation in soil, with total concentrations in the impact zone exceeding background levels: Cu by 4.5-fold, Zn by 2.5-fold, Pb by 2.9-fold, and Cd by 8.2-fold, consistent with previous research documenting HM accumulation from coal combustion emissions in the Rostov region [38,39,40]. However, total soil concentrations alone do not determine plant uptake; the chemical speciation of metals—their distribution among various soil fractions with different bioavailability—drives plant-available metal pools [41]. Root exudates form soluble complexes with HM, increasing the labile metal pool, while microbial activity in the rhizosphere generates redox gradients: the reduction of metal oxides releases trace metals, and the oxidation of organic matter mobilizes additional metals [10,42]. The analysis of metal bioavailability revealed critical differences between the background site and the impact zone. The contaminated area exhibited 10- to 100-fold increases in mobile metal form concentrations relative to background conditions. This poses significant risks to plant and microbial communities and facilitates metal entry into the food chain [43,44].

Dominant native plant species growing in contaminated areas can serve as valuable bioindicators and bioaccumulators of pollutants [45,46]. Plants show different strategies in response to HM pollution [47]. Metal-excluder plants restrict the translocation of HM into shoots and typically have lower HM concentrations in shoots [48]. BCF analysis demonstrated that *T. vulgare* was not a hyperaccumulator. Both BCF_shoot_ and BCF_root_ values for essential elements (Cu, Zn, and Ni) remained far from 1. Although Cd, Pb and Cr showed elevated BCF_shoot_ in the impact zone, absolute shoot concentrations remained far below hyperaccumulator thresholds (Cd: 0.92 mg/kg; Pb: 16.5 mg/kg; Cr: 35.7 mg/kg). Thus, *T. vulgare* behaves as a selective excluder with strong root-retention mechanisms rather than as a hyperaccumulator. Translocation factors revealed contrasting metal uptake strategies depending on the soil pollution level (Table 3). At the background site, *T. vulgare* maintained an excluder phenotype: Cu, Zn, Ni, and Pb displayed TF values below 1.0, indicating root-based retention mechanisms that prevented shoot accumulation. However, in the impact zone, metal translocation patterns shifted dramatically. Notably, excluder mechanisms for essential elements were strengthened: Cu TF decreased from 0.4 to 0.2, and Zn TF decreased from 0.6 to 0.5, suggesting that the plant enhanced the immobilization of beneficial metals under pollution stress. An adequate level of essential elements is maintained in metabolically active shoots, despite the high concentration in the soil. In stark contrast, toxic non-essential elements exhibited anomalous translocation: Cd TF increased by 5.6-fold and Pb TF increased by 6-fold, shifting from strong root retention to shoot accumulation. This breakdown of exclusion correlates directly with rhizosphere HM bioavailability: the 14-fold enrichment of mobile Cd and 22-fold enrichment of mobile Pb in the rhizosphere created biogeochemically available metal pools that overwhelmed the plant’s exclusion capacity, a pattern also reported in other heavily polluted environments [49,50]. Despite a 4.5-fold increase in soil Cu (from 33.4 to 150.2 mg kg^−1^), shoot Cu remained essentially unchanged (3.0 vs. 2.9 mg kg^−1^), while root Cu approximately doubled (7.9 to 15.1 mg kg^−1^). This preferential retention of Cu in roots demonstrates a robust root-to-shoot Cu transport limitation under HM stress, maintaining Cu homeostasis in metabolically active shoots. This paradox indicates that the Cu transport limitation is under strong physiological control. In contrast, Cd and Pb showed proportional increases in shoot accumulation with rising bioavailability. This thus highlights the effectiveness of Cu-specific homeostatic regulation in *T. vulgare*. HM exposure induced substantial oxidative stress in *T. vulgare*, manifested by elevated ROS production and membrane lipid peroxidation, which in turn negatively affected metabolic processes and cellular structure [51]. MDA content increased significantly in both roots and shoots at the polluted site. MDA accumulation reflects oxidative membrane damage, correlates with the disruption of chloroplast and cell membrane ultrastructure, and leads to degradation of leaf mesophyll ultrastructure [52]. Proline content was also higher in *T. vulgare* from the polluted area. Proline plays multiple roles in stress mitigation, functioning as a metal chelator and antioxidant [53], and additionally acting as a signaling molecule capable of inducing the expression of antioxidant enzyme genes [54,55]. The observed increase in proline thus reflects both osmoprotective and regulatory functions. Previous research demonstrated that HM induced proline accumulation in plants [14].

Plants activated multi-tiered antioxidant defenses in response to multi-metal stress. Glutathione content in shoots increased sharply (by 11%, relative to background site), consistent with its dual role as a direct ROS scavenger (via the ascorbate–glutathione cycle) and as a precursor for phytochelatins, which form metal–ligand complexes with HM [56,57]. In contrast, polyphenol content decreased despite the recognized ROS-scavenging functions of phenolics, likely reflecting metabolic depletion and reallocation under chronic stress [21,58]. Enzymatic antioxidant defenses were also strongly upregulated. POD activity in shoots increased by approximately 5-fold, making it the most responsive enzyme and confirming its value as a sensitive indicator of the onset of oxidative stress [59]. CAT activity in shoots rose by about 4.5-fold, whereas in roots, it declined, indicating the organ-specific allocation of defense: shoots prioritized CAT-mediated H_2_O_2_ detoxification where photosynthetic ROS production was highest. SOD activity increased and paralleled these trends, reflecting an enhanced dismutation of superoxide radicals. Overall, the stronger activation of antioxidant defenses in shoots is consistent with the preferential accumulation of toxic non-essential metals in aerial tissues.

Transmission electron microscopy showed severe organelle degradation in both the roots and shoots of plants from the contaminated area. Mitochondria displayed poorly defined cristae and reduced sizes, indicating impaired oxidative phosphorylation and ATP production capacity. The endoplasmic reticulum showed fragmentation with partial membrane ruptures, disrupting protein synthesis and trafficking. Chloroplasts exhibited thylakoid disorganization, plastoglobuli accumulation, and enlarged starch grains, reflecting photosynthetic dysfunction and accumulated photosynthate storage. The enlargement of the epiblem under stress may represent a protective adaptation to prevent excessive water loss or, alternatively, a consequence of reduced lateral growth in the primary cortex. Previous research demonstrated that HM stress affected root anatomy, damaged root cortical and epidermal cells, and inhibited water and nutrient absorption [60].

Despite pronounced ultrastructural damage at the cellular level, *T. vulgare* maintained remarkable organismal-level stability and high biomass productivity in contaminated soils. Sustained tissue growth and successful population establishment in polluted zones indicate that physiological compensation mechanisms prove sufficient for long-term plant survival. This organismal stability has significant practical implications for phytoremediation. *T. vulgare* combines several key advantages: high biomass productivity in contaminated soils; selective metal translocation to shoots; and native species status, eliminating cultivation costs. The plant’s combination of robust productivity, selective metal accumulation, native status, and establishment capacity in degraded habitats makes it uniquely suited for long-term, low-cost remediation of heavily polluted regions like the Novocherkassk impact zone.

## 4. Materials and Methods

### 4.1. Study Location and Environmental Conditions

**Figure 7:**
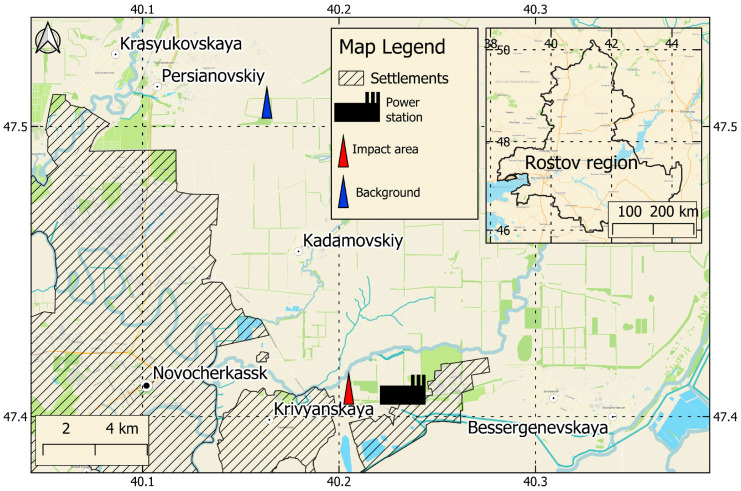
A schematic map with the location of monitoring plots.

The study was conducted in the impact zone of the Novocherkassk power station (impact area) and at a background site within the Persianovskaya protected steppe (background area) (Figure 7). The study site is located in the Rostov region, Russia. The Persianovskaya protected steppe (84 ha), situated approximately 12 km from the power station, was selected as a background area due to the absence of a direct anthropogenic impact. The impact area, situated approximately 1.9 km from the Novocherkassk power station, was subjected to a pollution load. The power station operates four chimneystacks, three with a height of 250 m and one with a height of 185 m, which represent the main pathways for atmospheric pollutant dispersion [61]. The study area is characterized by a humid continental climate (Dfa, Köppen–Geiger classification), with a mean annual temperature of approximately 11 °C, an average frost-free period of about 181 days and a mean annual precipitation of approximately 605 mm.

### 4.2. Soil and Plant Sampling and Determinations of Soil Physical and Chemical Properties

Soil and plant samples were collected in June, during the vigorous vegetation period. We established three sampling replicates at both the Persianovskaya protected steppe and at the polluted area near the Novocherkassk power station. In each replication, samples were collected at 10 randomly selected points. The approximate distance between sampling points were 3–5 m. These 10 samples were thoroughly mixed to form a composite sample. Soil samples were taken from the 0–20 cm layer. Rhizosphere and non-rhizosphere soil were separated by mechanical shaking. Soil that was freely shaken off the root surface was defined as non-rhizosphere soil, and soil adhering to roots after shaking was referred to as rhizosphere soil [62]. Root and shoot samples were oven-dried at 65 °C to a constant weight, ground and sieved prior to chemical analysis. Plant materials for biochemical analyses were immediately stored at −80 °C.

Soil pH was assayed by the potentiometric method in a soil-to-water suspension (1:2.5). Silt and clay fractions were determined by the pipette method. Carbonate content was assayed according to the Scheibler method. Exchangeable Ca^2+^ and Mg^2+^ were determined according to the ammonium acetate (NH_4_OAc) displacement method. The soil organic carbon content was assayed by the Walkley–Black wet oxidation method. The soil physicochemical properties of the study sites have been shown in Table 4.

### 4.3. Determination of HM in Soil and Rhizosphere

The total content of HM in the soil and rhizosphere was determined by X-ray fluorescence using a SPECTROSCAN MAX-GVM spectrometer, (Spectron, Saint Petersburg, Russia) [63]. Mobile forms were extracted from the soil using an ammonium acetate buffer solution with pH 4.8 and a soil/solution ratio of 1:10 for 18 h. The extracts were subsequently analyzed by atomic absorption spectrophotometry (Kvant-2, Kortec, Moscow, Russia) [6].

### 4.4. Determination of HM Content in T. vulgare and HM Translocation

Dried shoot and root samples (1 g) were ignited for 5 h at 500 °C in a muffle furnace and then cooled in a desiccator. Acid extractions of HM from ash were carried out by dissolving the ash in a 20% HCl solution [40]. HM content in the extract was measured using a Kvant-2 atomic absorption spectrometer (Kortek, Moscow, Russia).

The assessment of HM transfer from soil to plants and their translocation from roots into shoots were carried out based on the bioconcentration factor (BCF) and the translocation factor (TF) [64]. BCF was calculated as the ratio of the content of HM in the plant biomass (roots or aerial parts) to the concentration of HM in the soil:

BCF_root_ = C_root_/C_soil_

where C_root_ is the content of HM in the root (mg/kg) and C_soil_ is the total content of HM in the soil (mg/kg).

BCF_shoot_ = C_shoot_/C_soil_

where C_shoot_ is the content of HM in the shoot (mg/kg) and C_soil_ is the total content of HM in the soil (mg/kg).

TF was calculated as the ratio between the pollutant content in the stems and that in the roots (C_shoot_/C_root_):

TF = C_shoot_/C_root_

where C_shoot_ is the content of HM in the shoot (mg/kg) and C_root_ is the content of HM in the root (mg/kg).

### 4.5. Determination of Biochemical Parameters

#### 4.5.1. Determination of Malondialdehyde (MDA) Content

MDA content was assayed by the method of Hodges using 2-thiobarbituric acid [65]. Briefly, 2.5 mL of 0.6% thiobarbituric acid in 10% trichloroacetic acid was added to 0.5 g of plant biomaterial and was crushed in a mortar with liquid nitrogen. The mixture was heated at 100 °C for 15 min and then centrifuged at 10,000× *g* for 10 min. Absorbance was measured on a Beckman DU 800 spectrophotometer (Beckman Coulter, Inc., Brea, CA, USA) at 532 and 600 nm.

#### 4.5.2. Determination of Proline Content

For proline analysis, fresh tissue (0.5 g) was homogenized in 3% sulfosalicylic acid. The homogenate was centrifuged (16,000× *g*, 10 min, 4 °C), and 500 μL of plant extract from the shoots and roots was mixed with 500 μL of glacial acetic acid and 500 μL of ninhydrin reagent and then incubated for 30 min at 95 °C and cooled at room temperature [66]. Absorbance was measured at λ = 520 nm on a Beckman DU 800 spectrophotometer (Beckman Coulter, Inc., Brea, CA, USA). Proline content was calculated using a calibration curve.

#### 4.5.3. Determination of Antioxidant Enzyme Activity

To determine antioxidant enzyme activity, a 1 g sample of *T. vulgare* tissue was homogenized in liquid nitrogen in 9 mL of extraction buffer (0.1 M phosphate buffer, pH 7.5, containing 0.5 mM EDTA and 0.5 mM phenylmethylsulfonyl fluoride). The homogenates were centrifuged at 16,000× *g* at 4 °C for 20 min. The resulting enzyme extracts were used for the determination of antioxidant enzyme activity. SOD activity in the roots and shoots of *T. vulgare* was assessed by the inhibition of the photochemical reduction of nitroblue tetrazolium [67]. Guaiacol peroxidase activity was measured as the increase in absorbance at λ = 470 nm due to the formation of tetraguaiacol [68]. CAT activity was assessed by monitoring the decrease in H_2_O_2_ concentration [69]. Optical density was measured on a Beckman DU 800 spectrophotometer (Beckman Coulter, Inc., Brea, CA, USA).

#### 4.5.4. Determination of Polyphenol Content

The polyphenol content was assayed using the Folin–Ciocalteu reagent [70]. Pre-dried and ground plant material (40 mg) was extracted with 10 mL of 85% ethanol in the dark, at room temperature, for 24 h. The extract was centrifuged at 10,000× *g* for 5 min at 4 °C, and 1 mL of 80% alcohol extract was mixed with 2.5 mL of 10% Folin–Ciocalteu reagent and 2.5 mL of 7.5% sodium carbonate solution. The mixture was then incubated in the dark, at room temperature, for 20 min, and the absorbance was measured at λ = 765 nm on a Beckman DU 800 spectrophotometer (Beckman Coulter, Inc., Brea, CA, USA).

#### 4.5.5. Determination of Glutathione Content

Glutathione concentration was determined by reaction with Ellman’s reagent in a slightly alkaline medium, with the formation of a colored product [71]. Briefly, 20 μL of plant extract in 2% sulfosalicylic acid, 60 μL of glutathione reductase (3 U mL^−1^) and 60 μL of Ellman’s reagent (1.682 mM) were placed in a 96-well microplate. After exactly 30 s, 60 μL of β-NADPH (0.800 mM) was added. Optical density was measured at λ = 412 nm for two minutes every 30 s on a FLUOstar Omega microplate reader (BMG LABTECH, Ortenberg, Germany). The calculation was carried out using a calibration curve with glutathione as a standard.

### 4.6. Morphological and Ultrastructural Analysis

Microscopic examinations were carried out according to Fedorenko et al. [72]. Segments (2 × 2 mm) were cut from the absorption zone of roots and from the middle of the leaf. Samples were fixed in a 2.5% solution of glutaraldehyde in phosphate buffer (pH 7.4) for 4 h at room temperature. Roots and leaf segments were washed three times with phosphate buffer post-fixed with a 2% osmium tetroxide solution for 2 h. After washing with phosphate buffer, plant tissues were dehydrated stepwise in ethanol (30, 50, and 70%) and then kept in 70% ethanol containing uranyl acetate for 12 h at 4 °C. Dehydration was completed in 96 and 100% ethanol and then in 100% acetone (three changes, 15 min each). Samples were infiltrated with Epon resin in acetone at increasing resin concentrations and embedded in Epon. Polymerization was carried out in a thermostat at 37 °C for 1 day, 48 °C for 1 day and 60 °C for 2 days.

Semi-thin sections (0.5–1 μm) for light microscopy were prepared using a Leica EM UC6 ultramicrotome (Leica Microsystems, Vienna, Austria), stained with methylene blue and examined with a MIKMED-6 light microscope (LOMO, Saint Petersburg, Russia) at ×100 and ×400 magnification. Ultrathin sections (~100 nm) were examined using an FEI Tecnai G2 Spirit Bio-Twin transmission electron microscope (FEI, Eindhoven, The Netherlands). Digital images were acquired and processed using the Olympus Soft Imaging System (ITEM).

### 4.7. Statistical Analysis

An analysis of variance was performed using the Statistix 18.0 software, and the data of each parameter was analyzed separately. Comparisons between treatments were performed using *t*-test at *p* < 0.05. All figures were prepared using OriginPro 2021.

## 5. Conclusions

*T. vulgare* withstands chronic multi-metal contamination in the impact zone of the Novocherkassk coal-fired power station through rhizosphere-driven and plant-mediated adaptive mechanisms. HM stress triggered the strong activation of POD and CAT, which increased by approximately 5- and 4.5-fold in shoots, respectively, while glutathione and proline accumulated 3.4- and 1.7-fold, respectively.

The rhizosphere strongly amplified metal bioavailability, with mobile Cd and Pb enriched by 14-fold and 22-fold, respectively. At the same time, there was a stress-induced shift in translocation patterns, with non-essential toxic elements (Cd, Pb) increasingly transported to the shoots. These traits identify *T. vulgare* as a stress-tolerant species with phytoremediation potential for HM-polluted soils.

Future research should explore the rhizosphere microbial community composition and function in mediating *T. vulgare* selective metal tolerance, investigate the potential for repeated cycles of cultivation and metal removal to determine the actual remediation capacity and assess ecosystem-level impacts. Additionally, integrating multi-omics, rhizosphere microbiome analysis, and genetic approaches will be essential to unravel the regulatory network governing *T. vulgare* adaptations to HM-polluted soils.

## Figures and Tables

**Table 1:** Total and mobile forms of HM in the soil and rhizosphere (mg kg^−1^).

HM	Location	Total HM		Mobile Forms of HM	
		Soil	Rhizosphere	Soil	Rhizosphere
Copper	Background	33.41 ± 0.90 b	32.74 ± 0.92 b	0.08 ± 0.01 b	0.15 ± 0.02 b
	Impact area	150.2 ± 6.24 a	143.3 ± 4.16 a	18.4 ± 1.16 a	24.64 ± 0.47 a
Zinc	Background	78.23 ± 2.19 b	72.25 ± 2.02 b	2.65 ± 0.16 b	3.33 ± 0.21 b
	Impact area	196.7 ± 4.1 a	191.5 ± 5.4 a	21.83 ± 1.37 a	25.78 ± 0.60 a
Lead	Background	33.94 ± 1.46 b	27.27 ± 0.76 b	0.30 ± 0.02 b	0.72 ± 0.08 b
	Impact area	99.23 ± 3.98 a	95.64 ± 2.68 a	12.79 ± 0.80 a	15.82 ± 0.54 a
Nickel	Background	56.10 ± 1.51 b	54.25 ± 1.19 b	0.42 ± 0.02 b	0.37 ± 0.03 b
	Impact area	92.75 ± 1.74 a	95.26 ± 2.48 a	7.53 ± 0.47 a	9.33 ± 0.31 a
Manganese	Background	771.0 ± 10.8 b	795.6 ± 22.2 b	22.64 ± 1.42 b	38.95 ± 0.93 b
	Impact area	1063.9 ± 24.3 a	1054.3 ± 29.5 a	143.77 ± 8.91 a	184.25 ± 3.39 a
Cadmium	Background	0.35 ± 0.02 b	0.58 ± 0.1 b	0.013 ± 0.001 b	0.03 ± 0.02 b
	Impact area	2.88 ± 0.09 a	2.91 ± 0.08 a	0.75 ± 0.04 a	0.85 ± 0.05 a
Chromium	Background	118.0 ± 3.7 b	97.60 ± 2.34 b	1.52 ± 0.09 b	2.87 ± 0.13 b
	Impact area	176.9 ± 3.4 a	171.6 ± 4.8 a	14.28 ± 0.089 a	18.24 ± 0.70 a

Values are the means ± standard error of three replicates, and different lowercase letters within a column indicate significant differences at *p* ˂ 0.05 (*t*-test). Note: Background: Persianovskaya protected steppe; Impact area: impact zone of the Novocherkassk power station.

**Table 2:** HM in the shoots and in the roots of common tansy *(Tanacetum vulgare* L.) (mg kg^−1^).

Plant Part	Location	Copper	Zinc	Lead	Nickel	Manganese	Cadmium	Chromium
Shoot	Background	3.0 ± 0.32 a	7.7 ± 0.47 b	1.9 ± 0.14 b	1.7 ± 0.23 b	27.4 ± 0.89 b	0.12 ± 0.02 b	23.0 ± 0.79 b
	Impact area	2.9 ± 0.30 a	10.1 ± 0.78 a	16.5 ± 0.65 a	3.3 ± 0.20 a	40.9 ± 1.67 a	0.92 ± 0.06 a	35.7 ± 1.47 a
Root	Background	7.9 ± 0.42 b	12.0 ± 0.87 b	10.5 ± 0.62 b	3.7 ± 0.18 b	21.6 ± 0.89 b	0.15 ± 0.03 a	14.2 ± 0.82 b
	Impact area	15.1 ± 0.51 a	19.2 ± 1.4 a	13.3 ± 0.88 a	6.9 ± 0.43 a	27.3 ± 0.75 a	0.21 ± 0.03 a	32.4 ± 1.65 a

Values are the means ± standard error of three replicates, and different lowercase letters within a column indicate significant differences at *p* ˂ 0.05 (*t*-test). Note: Background: Persianovskaya protected steppe; Impact area: impact zone of the Novocherkassk power station.

**Table 3:** The bioconcentration factor and the translocation factor of HM by *T. vulgare*.

HM	Location	TF	BCF_root_	BCF_shoot_
**Copper**	Background	0.4	0.24	0.09
	Impact area	0.2	0.10	0.02
**Zinc**	Background	0.6	0.15	0.10
	Impact area	0.5	0.10	0.05
**Lead**	Background	0.2	0.31	0.06
	Impact area	1.2	0.13	0.17
**Nickel**	Background	0.5	0.07	0.03
	Impact area	0.5	0.07	0.04
**Manganese**	Background	1.3	0.03	0.04
	Impact area	1.5	0.03	0.04
**Cadmium**	Background	0.8	0.44	0.35
	Impact area	4.5	0.07	0.32
**Chromium**	Background	1.6	0.12	0.20
	Impact area	1.1	0.18	0.20

Background: Persianovskaya protected steppe; Impact area: impact zone of the Novocherkassk power station.

**Table 4:** Soil physicochemical properties of the study sites.

Location	pH	Clay Content	Silt Content	Organic Carbon Content	Calcium Carbonate Content	Exchangeable Ca^2+^	Exchangeable Mg^2+^
		%				cmol/kg	
**Background**	6.9–7.3	34–48	54–63	2.0–3.8	0.001–0.004	33.0–39.1	3.5–4.7
**Impact area**	6.9–7.5	23–29	50–56	1.8–3.1	0.02–0.1	28–33	2.1–4.1

Background: Persianovskaya protected steppe; Impact area: impact zone of the Novocherkassk power station.

## Data Availability

The original contributions presented in this study are included in the article. Further inquiries can be directed to the corresponding author.
